# Magnetic and Golden Yogurts. Food as a Potential Nanomedicine Carrier

**DOI:** 10.3390/ma13020481

**Published:** 2020-01-19

**Authors:** Víctor Garcés, Ana González, Laura Sabio, Carmen M. Sánchez-Arévalo, Natividad Gálvez, José M. Dominguez-Vera

**Affiliations:** Departamento de Química Inorgánica, Instituto de Biotecnología, Facultad de Ciencias, Universidad de Granada, 18071 Granada, Spain; vigaro@ugr.es (V.G.); agongar@ugr.es (A.G.); laurasabio@ugr.es (L.S.); carsana5@upv.es (C.M.S.-A.);

**Keywords:** yogurt, gold nanoparticles, magnetic nanoparticles, functional food

## Abstract

Yogurt is one of the most emblematic and popular fermented foods. It is produced by the fermentation of milk lactose by bacteria such as *Streptococcus thermophilus* and *Lactobacillus acidophilus*. Magnetic (MNPs) and gold nanoparticles (AuNPs) were incorporated into the exopolysaccharides (EPSs) of these bacteria. The functionalized bacteria were characterized by UV-vis spectroscopy and transmission electron microscopy. A large number of MNPs and AuNPs were bound to the bacterial EPS. Interestingly, the nanoparticles’ (NPs) presence did not affect the bacteria’s capacity to ferment milk and to produce magnetic and golden yogurts. Magnetic and golden yogurts represent the perfect combination of emblematic food and nanoparticles and have a range of potential biomedical applications: use in iron-deficiency anemia, diagnosis and hyperthermia treatment of appropriate digestive diseases, and interest in glamour cuisine.

## 1. Introduction

Yogurt is one of the oldest and most popular fermented foods worldwide. It is produced by the bacterial fermentation of milk [1]. Genuine bacteria culture consists of a mixture of *Lactobacillus acidophilus* and *Streptococcus thermophilus*. These bacteria ferment milk lactose to produce lactic acid, which denatures milk proteins at temperatures of around 40 °C to give yogurt [2].

Yogurt keeps for longer than milk, and together with its healthy properties [3,4,5] and great taste, it is an emblematic food that has evolved over centuries into today’s commercial yogurt industry. This year marks the 100th anniversary of the first industrialized production of yogurt, attributed to Isaac Carasso in 1919 in Barcelona [6].

We have studied whether yogurt may serve as a matrix for the incorporation of therapeutic substances. This novel approach aims to give to food additional properties that are beneficial for health. With this aim, we have incorporated two of the most genuine materials in nanomedicine, maghemite (MNPs) and gold nanoparticles (AuNPs). MNPs are of paramount importance as diagnostic tools in magnetic resonance imaging [7], as mediators for hyperthermia cancer treatment [8] and as drug delivery vehicles [9,10,11]. AuNPs have a large number of applications thanks to their unique optical, electrical, and photothermal properties, which are particularly useful, for instance, in cancer treatments [12,13,14].

We have previously demonstrated that iron oxide and gold nanoparticles can be incorporated within the exopolysaccharide matrix (EPS) of some probiotic bacteria leading to live, hybrid probiotic-NPs materials [15,16]. EPS are natural polysaccharides secreted by some bacteria. They play a crucial role in bacterial surface adhesion, an essential step for colonization [17,18]. EPS of probiotic bacteria present in human gut microbiota represent an extraordinarily effective platform for the aggregation of nanoparticles of different sizes and shapes [19].

Moreover, we found that probiotics serve as oral carriers of MNPs as they overcome the intestinal barrier and reach the intestine where iron absorption takes place [20,21]. *L. fermentum*–MNPs are an excellent candidate for the treatment of iron-deficiency anemia.

Against this background, we addressed the incorporation of MNPs and AuNPs within yogurt-producing bacteria. In the case of AuNPs, we also tried to use the bacteria as bioreactors for the direct synthesis of AuNPs from metal ions. The latter application is a nice example of green chemistry that avoids the use of toxic chemicals, high pressures, and high temperatures [22,23]. The biosynthesis of metallic nanoparticles using microorganisms as reducing agents has gained popularity in recent decades as it presents several advantages over traditional synthetic methods [22,23]. There are other microorganisms with this reducing capacity, including several probiotic bacteria such as *Lactobacillus fermentum* [16].

Combining probiotic bacteria’s capacity to nest in different tissues of the gastrointestinal tract and the interesting optical and magnetic properties of AuNPs and MNPs, we intend to produce new functional foods for biomedical applications [24,25,26,27].

## 2. Materials and Methods

### 2.1. Materials

All reagents were purchased at Sigma Aldrich (San Luis, MI, USA). Live/dead bacterial viability kits SYTO9 and propidium iodine were purchased at ThermoFisher (Waltham, MA, USA). UV-vis spectra were recorded at a Unicam UV 300 Thermo Spectronic spectrophotometer. Transmission Electron Microscopy were obtained in a LIBRA 120 PLUS microscope (Carl Zeiss SMT), and High-Angle Annular Dark Field (HAADF) images were obtained in a FEI TITAN G2 microscope. Laser confocal Images were obtained in a confocal microscope Leica DMI6000. All devices were from Centro de Instrumentación Científica (CIC) of the University of Granada.

### 2.2. Synthesis of Maghemite Nanoparticles

Maghemite nanoparticles (MNPs) were synthesized according to Massart’s method by coprecipitation of Fe(II) and Fe(III) salts in a stoichiometry of 0.5 [28]. By adjusting the pH to 11 with 3 M Sodium hydroxide (NaOH) and ionic strength with 1 M Sodium nitrate (NaNO_3_), the average size of the resulting magnetite nanoparticles is 10 nm. After oxidation of magnetite to maghemite with 1 M Perchloric acid (HClO_4_), a colloid of maghemite nanoparticles stable at pH 2 was obtained.

### 2.3. Synthesis of Spherical Gold Nanoparticles

Spherical gold nanoparticles (AuNSs) were synthesized following a previously reported protocol [29]. Five mL of a 1.0 mM Gold (III) chloride trihydrate (HAuCl_4_·3H_2_O) solution in water was stirred and heated to boiling on a hot plate. After the solution began to boil, 500 µL of a 38.8 mM Sodium citrate (Na_3_C_6_H_5_O_7_) solution in water was added. The mixture was boiled and stirred continuously for about 10 min until it was a deep red color. The solution was cooled to room temperature. AuNSs were characterized by UV-vis spectroscopy and transmission electron microscopy (TEM).

### 2.4. Synthesis of Gold Nanorods

To prepare Gold Nanorods (AuNRs), a seed-mediated growth method was carried out [30]. Solutions of HAuCl_4_·3H_2_O 1 mM, Silver nitrate (AgNO_3_) 8 mM, Ascorbic Acid (C_6_H_8_O_6_) 78.8 mM, and Sodium borohydride (NaBH_4_) 10 mM were prepared at room temperature. The NaBH_4_ solution was ice-cooled after preparation. Solutions of Cetyl trimethylammonium bromide (CTAB) 0.2 M and (11-Mercaptoundecyl)-*N*,*N*,*N*-trimethylammonium bromide 92 mM were separately prepared by heating at 50 °C and stirring until dissolved and then cooled to room temperature. All the solutions were prepared in Mili-Q ultrapure water. Gold seed synthesis was as follows: 5 mL of CTAB 0.2 M, 2.5 mL of HAuCl_4_ 1 mM, and 0.6 mL of ice-cold NaBH_4_ 10 mM were mixed and stirred for 2 min at 25 °C. When seeds were formed, the solution color changed from yellow to slightly brown. AuNRs synthesis was as follows: 5 mL of the 0.2 M CTAB solution was placed in a flask previously set in an oil bath at 30 °C under stirring at 180 rpm. 5 mL of HAuCl_4_ 1 mM, 70 µL of ascorbic acid 78.8 mM, and 100 µL of AgNO_3_ 8 mM were added in this order to the CTAB solution. The mixture was completed with 160 µL of the seed solution. The completed mixture was kept stirring at 180 rpm and 30 °C for 48 h. Due to CTAB antibacterial activity [31], it was replaced by MUTAB ((11-Mercaptoundecyl)-*N*,*N*,*N*-trimethylammonium bromide). For this, gold nanoparticles with CTAB as cap ligand were collected twice by high-speed centrifugation at 13,000 rpm for 10 min, dissolved in 1 mL of Mili-Q ultrapure water and 1 mL of MUTAB 92 mM, and stirred mildly. Gold nanoparticles were collected again by high-speed centrifugation at 13,000 rpm for 10 min 48 h later, and the pellet was dissolved in 1 mL of Mili-Q ultrapure water. The AuNRs were characterized by UV-Vis spectroscopy and TEM.

### 2.5. Synthesis of Gold Nanoprisms

Gold nanoprisms (AuNPRs) were synthesized following a previously reported protocol [32]: 100 mL of HAuCl_4_ 2 mM and 120 mL of fresh Sodium thiosulfate (Na_2_S_2_O_3_) 0.5 mM, both prepared in Mili-Q ultrapure water, were mixed and stirred gently at 15 °C. After 9 min (“seed” formation), an extra 50 mL of fresh Na_2_S_2_O_3_ 0.5 mM was added. Growth mixture was left overnight at 15 °C under mild stirring conditions. The AuNPRs were characterized by UV-vis spectroscopy and TEM.

### 2.6. Grafting Maghemite Nanoparticles to Bacteria

For the preparation of bacteria labeled with maghemite nanoparticles (MNPs–ferment), *Streptococcus thermophilus* and *Lactobacillus acidophilus*, supplied by Biosearch S.A, were grown together in a synthetic bacterial growth medium at 37 °C on an orbital shaker for 24 h with an initial concentration of 1 mg bacteria in 1 mL of medium. The synthetic growth medium consisted of 5.0 g L^−1^ of sodium phosphate dibasic (Na_2_HPO_4_), 6.0 g L^−1^ of monopotassium phosphate (KH_2_PO_4_), 2.0 g L^−1^ of tris-ammonium citrate (C_6_H_14_N_2_O_7_), 50.0 g L^−1^ of sucrose (C_12_H_22_O_11_), 1.0 g L^−1^ of magnesium sulfate (MgSO_4_), and 10 mL of a trace elements solution (consisting of the following: 2.0 g L^−1^ of manganese sulfate (MnSO_4_), 1.0 g L^−1^ of cobalt chloride (CoCl_2_), and 1.0 g L^−1^ of zinc chloride (ZnCl_2_) dissolved in 0.1 M hydrochloric acid (HCl) solution). The medium had an initial pH 6.7 and was sterilized at 121 °C. Bacteria were centrifuged at 3000 *g* for 5 min and washed with distilled water. Then, an acid solution (pH 2) of MNPs (66.6 μL, 0.95 M) was added to the bacteria in an ice bath and mixed. The solution was diluted to 1 mL with distilled water. MNPs–ferment was collected at 100 g, 20 min.

MNPs–ferment characterization was carried out by TEM as previously reported [11]. To do this, a drop of MNPs–ferment was placed onto a carbon-coated Cu grid (200 mesh) specific for TEM. The grid was blotted with filter paper. Electron micrographs were taken with a Philips CM-20 HR electronic analysis microscope operating at 200 keV. The grid was observed in a FEI TITAN G2 microscope, and the characterization was made by Scanning Electron Microscopy (HAADF-STEM), X-ray scattered energy (EDX map), and High-Resolution Transmission Electron Microscopy (HRTEM).

### 2.7. Grafting Gold Nanoparticles to Bacteria

Seventy-seven mg of a mixture of *Streptococcus thermophilus* and *Lactobacillus acidophilus* was cultured in 10 mL of synthetic bacterial growth medium at 37 °C on an orbital shaker for 24 h. This initial culture was divided into three parts that were collected by centrifugation at 3000 *g* for 10 min. To each bacterial pellet, 2 mL of a colloid with each of the morphologies of AuNPs (AuNSs, AuNRs, and AuNPRs) were added. After addition of AuNPs, labeled bacteria (AuNSs–ferment, AuNRs–ferment, and AuNPRs–ferment) were collected at 100 g, 20 min and were characterized by UV-vis spectroscopy and TEM.

### 2.8. Biosynthesis of Gold Nanoparticles by Bacteria

*Streptococcus thermophilus* and *Lactobacillus acidophilus*, supplied by Biosearch S.A. (Granada, Spain), were grown together as it was previously explained in Section 2.6. A 1-mM aqueous solution of HAuCl_4_ 3H_2_O in Type 1 Milli-Q ultrapure water was prepared; 2.5 mL of the gold solution were added to 7.5 mL of bacteria culture to give a final gold concentration of 0.25 mM. The mixture was incubated at 37 °C without stirring for 2 and 24 h to form labeled bacteria by autoreduction of Au(III). The synthesized AuNPs over bacteria (SAuNPs–ferment) were obtained by centrifugation at 100 g for 30 min and were characterized by UV-vis spectroscopy and TEM. Controls consisting of bacterial supernatants in the absence of *Streptococcus thermophilus* and *Lactobacillus acidophilus* were also testing for reducing activity.

### 2.9. Gold Reducing Bacteria Viability

Quantification of gold-reducing bacteria proliferation was performed by using the live/dead bacterial viability kits SYTO9 (green) and propidium iodine (PI) (red) (ThermoFisher) for confocal laser scanning microscopy. Following the manufacturer´s instructions, the molar ratio between SYTO9 and PI in the mixture was 1:6. A drop of the labeled bacteria was deposited onto a polylysine glass and observed in a confocal microscope Leica DMI6000, counting the number of live (green) and dead (red) bacteria in a batch of three experiments with the software Image-Pro Plus 6.0 (Media Cybernetics, Inc., Rockville, MD, USA). The average live/dead ratio was used to quantify the bacterial viability.

### 2.10. Yogurt Production by Labeled Bacteria

Each of the bacterial preparations, carrying the different types of metal nanoparticles MNPs–ferment, AuNSs–ferment, AuNRs–ferment, AuNPRs–ferment, and SAuNPs–ferment were separately added to fresh milk (Puleva Fresca^®^, Granada, Spain) at a temperature around 40 or 42 °C (7 mg of sample per each mL of milk). The fermentation progress was controlled by continuously measuring pH. The end of the fermentation was placed at pH 4.2–4.5.

## 3. Results and Discussion

**Scheme 1:**
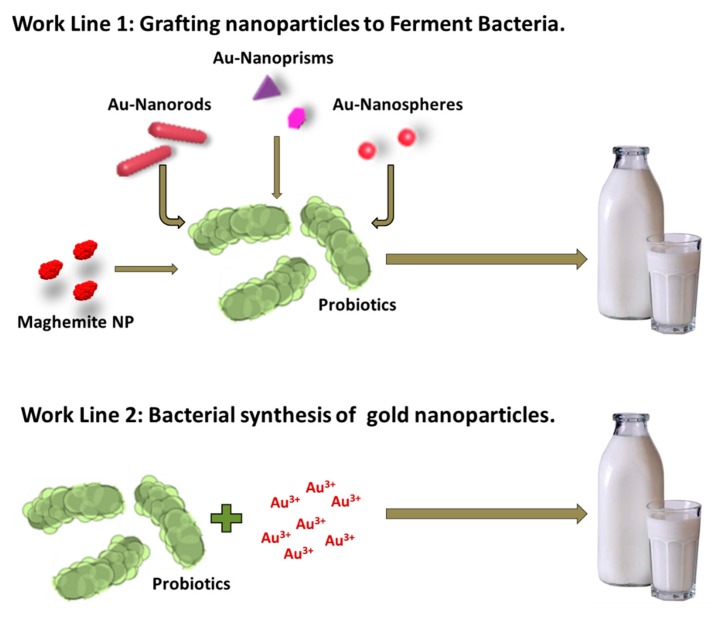
Schematic representation of the two experimental approaches followed to produce magnetic (MagYogurt) and golden yogurts (YogAur).

We followed two different approaches to produce live, hybrid probiotic-NP materials (Scheme 1). Firstly, previously synthesized maghemite and gold nanoparticles (spheres, rods, and prisms) were separately grafted to a mixture of *Streptococcus thermophilus* and *Lactobacillus acidophilus*. Secondly, the same mixture was used as a reducing agent to directly incorporate the gold nanoparticles onto their surface (biosynthetic approach). We then tested all the hybrid materials’ ability to produce yogurt. We have named these hybrid materials magnetic (MagYogur) or golden yogurts (YogAur).

### 3.1. Grafting Maghemite Nanoparticles to Bacteria

Following our protocol reported previously incorporating MNPs to probiotic bacteria [15], 10-nm MNPs were added to a fermentation culture containing a mixture of *Streptococcus thermophilus* and *Lactobacillus acidophilus.* The adhesion of MNPs to probiotics (MNPs–ferment) was confirmed through microscopic techniques such as TEM and HAADF-STEM. Metal content was analyzed by EDX spectroscopy.

**Figure 1:**
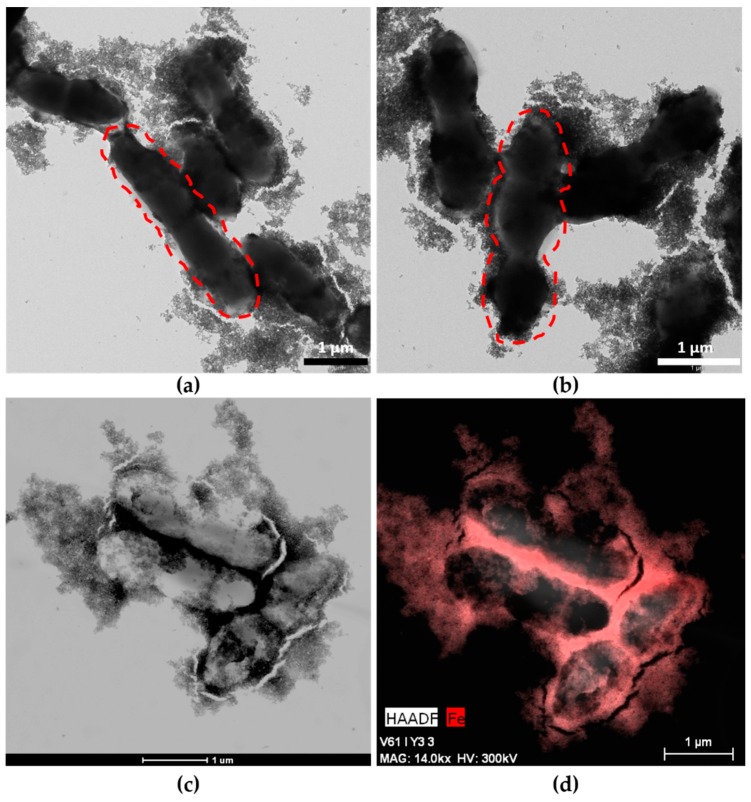
Maghemite nanoparticles (MNPs)–ferment observed by TEM and High-Angle Annular Dark Field-Scanning Transmission Electron Microscopy (HAADF-STEM): (**a**) TEM image of MNPs–ferment showing the presence of *Lactobacillus acidophilus* (dashed lines) with MNPs adhered to the exopolysaccharides (EPS); (**b**) TEM image of MNPs–ferment showing the presence of *Streptococcus thermophilus* (dashed lines) with MNPs adhered to the EPS; (**c**) HAADF-STEM image of the MNPs–ferment sample; and (**d**) EDX map of the MNPs–ferment sample with iron highlighted in red.

TEM images (Figure 1a,b) show MNPs adhered to the entire surface of both strains of bacteria, and these hybrids appeared very similar to systems obtained previously with other probiotics [15]. MNPs adhere to bacterial EPS to produce the MNPs–ferment. EPSs are the main component of the bacterial biofilm, an extrabacterial conglomeration of products surrounding the bacterial wall. The different morphologies of *Streptococcus thermophilus* and *Lactobacillus acidophilus* allowed us to confirm the presence of both bacteria in the ferment and the adhesion of MNPs to both probiotics (Figure 1a,b). *Lactobacillus* bacteria are large bacilli (2–5 µm), whereas *Streptococcus* is invariably smaller (1–2 µm) and grows through chain or pair formation. The high number of MNPs adhered to the EPS was also observed by HAAD-STEM microscopy (Figure 1c), and an EDX map of the MNPs–ferment sample confirmed the presence of iron in the bacterial EPS (Figure 1d).

### 3.2. MagYogurt

To evaluate the viability of the bacteria in the MNPs–ferment system, we decided to test their main activity: the fermentation of milk to produce yogurt. This process is considered a spontaneous lactic fermentation which requires both lactic acid bacterium (LAB) strains, i.e., *Streptococcus thermophilus* and *Lactobacillus acidophilus*. *Streptococcus thermophilus* is classified as a type A LAB, which causes an initial decrease in pH, and is associated with CO_2_ production and the appearance of anaerobic conditions. After that, type B and C LABs, for example *Lactobacillus acidophilus*, complete the fermentation process at pH 4.5 thanks to their greater resistance to acidic conditions [33].

**Figure 2:**
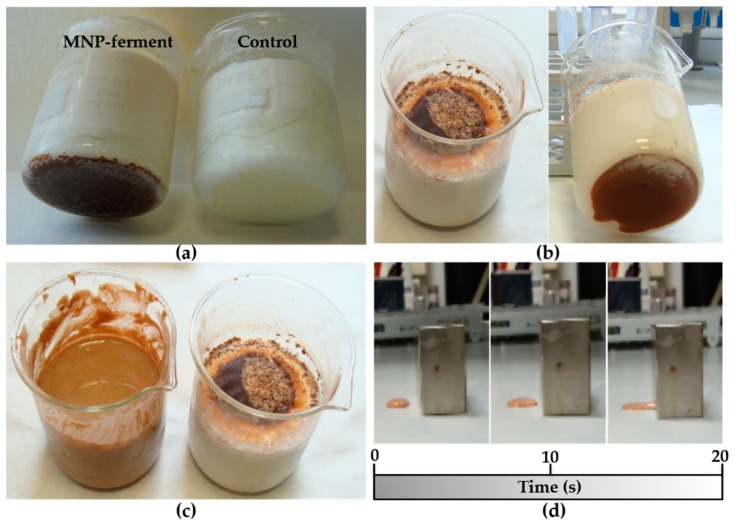
Photographs of different MagYogurt preparations: (**a**) MagYogurt obtained by MNPs–ferment (left) compared with the control produced from a ferment in the absence of magnetic nanoparticles (right); (**b**) MagYogurt obtained using a combination of both MNPs–ferment and MNPs–bacteria; (**c**) Liquid MagYogurt obtained using a 1.2 T magnet (left) compared to a sample prepared without a magnet; (**d**) movement of the liquid MagYogurt attracted by a 1.2 T magnet after 0, 10, and 20 s of exposition to the magnetic force.

In the initial experiment, MNPs–ferment was added to milk at 40–42 °C. After 8 h of incubation, we obtained yogurt with a pH of 4.5 and similar organoleptic properties to those of the control (ferment without magnetic nanoparticles) (Figure 2a). In a second experiment, we tried to increase the number of MNP-containing probiotics in the yogurt. Hence, we decided to enrich this yoghurt by incorporating a new probiotic: *Lactobacillus fermentum* bacteria labelled with maghemite nanoparticles (MNPs–bacteria). The addition of MNPs–ferment and MNPs–bacteria systems to milk produced yogurt (Figure 2b). However, in both experiments (Figure 2a,b), magnetic nanoparticles accumulated at the bottom of the beaker, so the magnetic material was not distributed homogeneously throughout the yogurt. To resolve this issue, we stirred the mixture and placed a 1.2 T magnet over the beaker during the fermentation. This yielded a liquid yogurt with homogeneously distributed magnetic material (Figure 2c). When a few drops of the liquid yogurt were placed on a surface in close proximity to the 1.2 T magnet, the MagYogurt flowed along the surface and reached the magnet in just 20 s (Figure 2d and Supplementary Materials Video S1). These results show that it is possible to produce MagYogurt at room temperature.

### 3.3. Grafting Gold Nanoparticles to Bacteria

**Figure 3:**
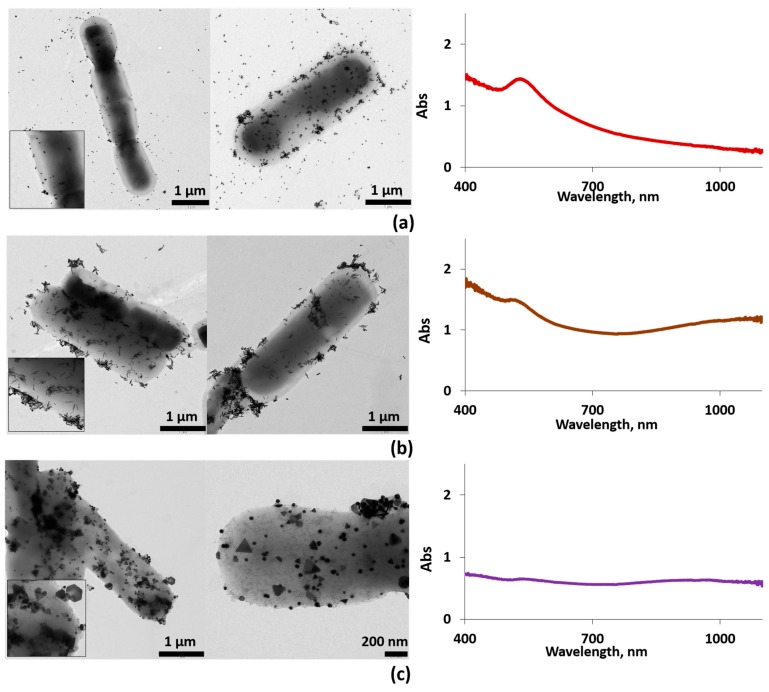
Left column: typical EM images for AuNSs–ferment (**a**), AuNRs–ferment (**b**), and AuNPRs–ferment (**c**). Several EM images are shown for each sample. Insets: zoom of the EM images. Right column: the corresponding UV-vis spectra of each sample, AuNSs–ferment (**a**), AuNRs–ferment (**b**), and AuNPRs–ferment (**c**).

AuNPs of different morphologies were synthesized following protocols reported previously; spheres (AuNSs) [29], rods (AuNRs) [30,31], and prisms (AuNPRs) [32]. These AuNPs were separately added to *Streptococcus thermophilus* and *Lactobacillus acidophilus* ferment in order to obtain labeled bacteria (AuNSs–ferment, AuNRs–ferment, and AuNPRs–ferment). Samples were characterized by UV-vis and TEM (Figure 3).

The TEM images reveal that large numbers of the AuNPs clearly adhered to the bacterial EPS in the form of small aggregates while very few AuNPs remained unattached to the bacterial EPS (Figure 3). Furthermore, the AuNPs did not undergo any changes in size or shape after interacting with both bacteria.

The UV-vis spectrum for the AuNSs–ferment showed the typical AuNSs surface plasmon resonance (SPR) band for an average size of 13 nm (Figure S1), centered at 530 nm (Figure 3b). In the case of AuNRs and AuNPRs, the SPR band splits into two bands; the first corresponds to the transverse mode centered at 530 nm, and the second one at lower energies is the resonance of the longitudinal mode (varying from 700 to 1000 nm). For AuNRs, this second band depends on the rods’ length-to-width ratio, whereas for AuNPRs, it can reach values of 1100 nm, which lies in the near-infrared region (Figure S1) [12,34,35,36]. In the case of the AuNRs–ferment and AuNPRs–ferment hybrids, the UV-vis spectra showed a broad band shifted to 900 nm for AuNPRs–ferment (Figure 3c) and to 1100 nm (near-infrared light (NIR)) for AuNRs–ferment (Figure 3b). The shift to lower energies of these bands is probably a consequence of the strong interparticle interactions present in the small aggregates formed at the bacterial EPS. We have previously observed this phenomenon when depositing AuNPs directly onto EPS isolated from probiotic bacteria [19]. Therefore, we can infer that the formation of gold aggregates is responsible for the shift in typical AuNPs SPR bands to lower energies, thereby improving the optical properties of the AuNPs–ferment compared to isolated gold nanoparticles. This may be of interest when exploring the clinical application of these systems because NIR light can penetrate much deeper inside the body to reach AuNPs.

### 3.4. YogAur

**Figure 4:**
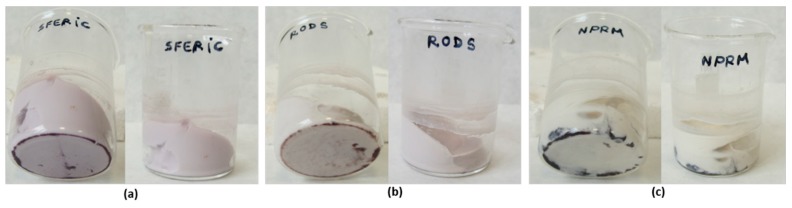
Photographs of YogAur obtained by AuNSs–ferment (**a**), AuNRs–ferment (**b**), and AuNPRs–ferment (**c**).

Following the same synthetic protocol, AuNSs–ferment, AuNRs–ferment, and AuNPRs–ferment were separately added to milk at 40–42 °C. All the ferments could produce yogurt after 8 h of incubation with a final pH of 4.2–4.5 (Figure 4). These results confirm the bacteria were still alive after being grafted with AuNPs of different morphologies. In addition, the nanoparticle morphologies were not altered during yogurt production because the characteristic color of gold nanoparticles was visible in the final fermentation product (Figure 4).

### 3.5. Biosynthesis of Gold Nanoparticles by Bacteria

In view of our previous results with *Streptococcus thermophilus* and *Lactobacillus acidophilus* as AuNP carriers, we decided to examine the possibility of using them as reducing agents in order to synthesize and incorporate AuNPs directly on their surface. The ferment was incubated with a 1 mM gold salt solution (HAuCl_4_ 3H_2_O). An optimal incubation time was selected based on some previously published results which demonstrated that gold ions can act as antimicrobial agents at a concentration 1000 times less than the one we were using [37,38] and that they are toxic for bacterial strains such as *Escherichia*, *Staphylococcus*, and *Salmonella* [39]. Taking this into account, all our experiments were performed at two different incubation times: a short period of 2 h and a longer one of 24 h. The formation of AuNPs under these conditions was followed by confirmation with UV-vis spectroscopy and TEM. The optimal conditions for synthesizing AuNPs on bacteria (SAuNPs–ferment) were 37 °C and with no stirring. Controls consisting of bacterial culture supernatants prepared without *Streptococcus thermophilus* and *Lactobacillus acidophilus* but under the same chemical conditions did not contain any gold nanoparticles, which indicates that the bacteria is crucial to synthesize AuNPs.

**Figure 5:**
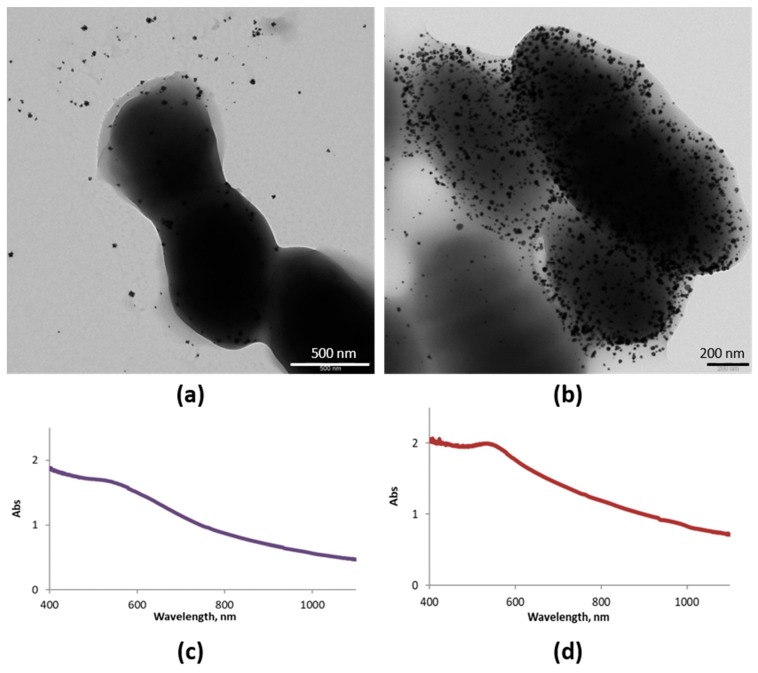
Synthesized AuNPs (SAuNPs)–ferment data obtained by UV-vis and TEM: (**a**) TEM images for SAuNPs–ferment obtained after 2 h (**a**) and 24 h (**b**) of incubation. UV-vis spectra for SAuNPs–ferment obtained after 2 h (**c**) and 24 h (**d**) of incubation.

Figure 5 shows TEM images of samples obtained after 2 and 24 h of incubation of Au (III) and the ferment. Spherical AuNPs were obtained, the amount of which increased with the incubation time (Figure 5a,b). UV-vis spectra confirmed the spherical morphology in both cases as they presented the typical SPR band (Figure 5c,d). However, the band shifted slightly to lower energies, as it is centered at 540 nm instead of 530 nm, probably because of the aggregation of AuNPs over the bacterial EPS. These results confirm that *Streptococcus thermophilus* and *Lactobacillus acidophilus* biosynthesize AuNPs at their surfaces.

**Figure 6:**
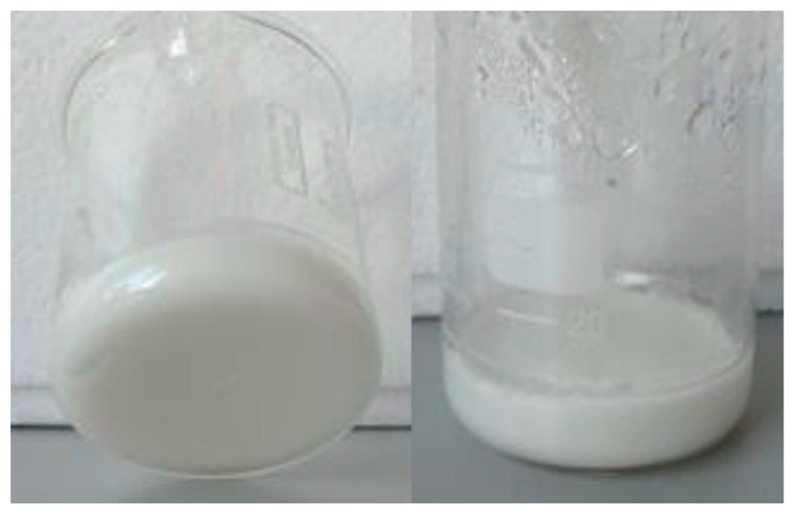
YogAur produced with SAuNPs–ferment obtained after 2 h of incubation.

However, when the 2 h and 24 h SAuNPs–ferments were added to milk at 40–42 °C to confirm the bacteria’s viability and fermentative capacity, we only observed yogurt production for the sample containing the 2 h SAuNPs–ferment (Figure 6). This indicates that gold ions have a toxic effect on probiotic bacteria and that this effect is greater after longer incubation periods. Hence, the 24 h SAuNPs–ferment bacteria did not present any metabolic activity; that is, they are nonviable bacteria.

### 3.6. Viability of Gold-Reducing Bacteria

**Figure 7:**
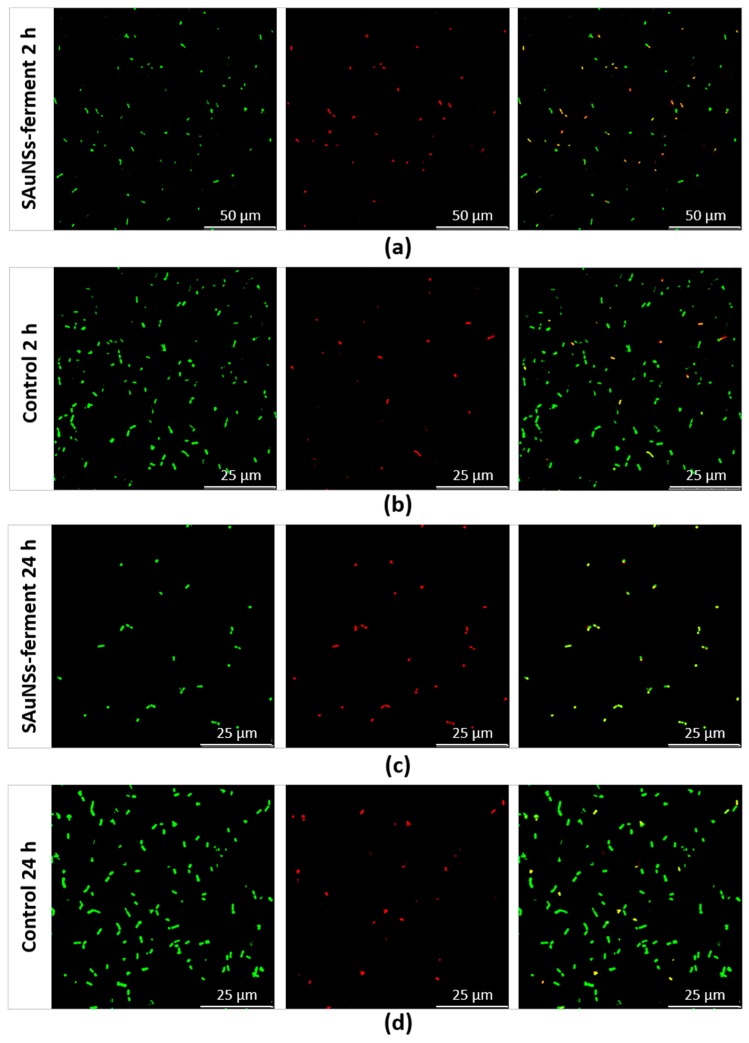
Confocal laser scanning microscopy images of SAuNPs–ferment after 2 h (**a**) and 24 h (**c**) incubations compared to control cultures lacking gold ions at the same times: 2 h (**b**) and 24 h (**d**). From left to right for each sample: all bacteria (spots stained green by SYTO9), dead bacteria (spots stained red by propidium iodide (PI)) and a merge of both images, where green and yellow spots corresponds to live and dead bacteria, respectively.

To confirm the reduced viability of the sample incubated for 24 h compared to the 2 h sample, we performed a final experiment using confocal laser scanning microscopy (CLSM). We used a standard live/dead test consisting of SYTO9 and propidium iodide (PI) dyes. Both of these compounds can bind to the nucleic acids of bacteria, but they have different capacities to penetrate inside them. While PI (red) only penetrates in nonviable bacteria with damaged membranes, SYTO9 (green) labels all bacteria. Consequently, CLSM reveals red fluorescent spots corresponding to dead bacteria, while live bacteria appear as green spots in the merged image (red + green channel). CLSM images obtained for SAuNPs–ferments are shown in Figure 7, where we compare them against two control cultures (incubated for 2 and 24 h) devoid of any gold ions. We quantified the viability of each sample by counting the number of red spots in the red channel and of green spots in the merged channel. The control samples reached viabilities of 93.7% and 98.2% after 2- and 24-h incubations, respectively. However, for the SAuNPs–ferment samples, the viability after 2-h incubation was 42.8%, which was five times higher than for the 24 h ferment of which viability was only of 9%. The toxic effect is therefore much greater after longer exposures. Further works are needed to reveal the chemical species that causes this toxicity. It can be speculated that the toxicity could be originated by the gold nanoparticles that progressively accumulate at the bacterial surface or by gold ions before reduction. In this sense, Bansal et al. have reported that some microorganisms reduce silver and gold ions (producing the metal nanoparticles) in response to remedying the toxicity of these metal ions [22,23]. The biosynthesis of gold nanoparticles by the ferment therefore requires a metabolic effort of the bacteria to reduce gold ions in such a way that SAuNPs–ferment is not more functional and is unable to ferment the milk to produce yogurt.

## 4. Conclusions

Magnetic and golden yogurts were produced by incorporating maghemite and gold nanoparticles within the EPS of *Streptococcus thermophilus* and *Lactobacillus acidophilus*. The functionalized bacteria were still alive and able to ferment milk and to produce yogurt. This type of product represents a new concept in functional food based on the health benefits of yogurt and the potential applications of magnetic and gold nanoparticles in biomedicine. Additionally, these yogurts can be considered glamorous foods with healthy properties and could be of interest in modern cuisine.

## Supplementary Materials

The following are available online at https://www.mdpi.com/1996-1944/13/2/481/s1, Figure S1: TEM images of AuNPs before adhesion to bacteria and their typical SPR bands; Video S1: Movement of the magnetic liquid yogurt attracted by a 1.2 T magnet.

## References

[1] Leroy F., De Vuyst L. (2004). Lactic acid bacteria as functional starter cultures for the food fermentation industry. Trends Food Sci. Technol..

[2] Ustunol Z. (2013). Development and Manufacture of Yogurt and Other Functional Dairy Products (2010), edited by F.Yildiz, CRC Press (Taylor and Francis Group), Boca Raton, FL, USA. ISBN 978-1-4200-8207-4. Int. J. Dairy Technol..

[3] Rizkalla S.W., Luo J., Kabir M., Chevalier A., Pacher N., Slama G. (2000). Chronic consumption of fresh but not heated yogurt improves breath-hydrogen status and short-chain fatty acid profiles: A controlled study in healthy men with or without lactose maldigestion. Am. J. Clin. Nutr..

[4] Labayen I., Forga L., Gonzalez A., Lenoir-Wijnkoop I., Nutr R., Martinez J.A. (2001). Relationship between lactose digestion, gastrointestinal transit time and symptoms in lactose malabsorbers after dairy consumption. Aliment. Pharmacol. Ther..

[5] Pelletier X., Laure-Boussuge S., Donazzolo Y. (2001). Hydrogen excretion upon ingestion of dairy products in lactose-intolerant male subjects: Importance of the live flora. Eur. J. Clin. Nutr..

[6] Tribune T.F.I.H. (2005). Danone Plays Down Rumor of a Takeover by PepsiCo.

[7] Pellico J., Ruiz-Cabello J., Fernández-Barahona I., Gutiérrez L., Lechuga-Vieco A.V., Enríquez J.A., Morales M.P., Herranz F. (2017). One-Step Fast Synthesis of Nanoparticles for MRI: Coating Chemistry as the Key Variable Determining Positive or Negative Contrast. Langmuir.

[8] Beola L., Asín L., Fratila R.M., Herrero V., de la Fuente J.M., Grazú V., Gutiérrez L. (2018). Dual Role of Magnetic Nanoparticles as Intracellular Hotspots and Extracellular Matrix Disruptors Triggered by Magnetic Hyperthermia in 3D Cell Culture Models. ACS Appl. Mater. Interfaces.

[9] Frimpong R.A., Hilt J.Z. (2010). Magnetic nanoparticles in biomedicine: Synthesis, functionalization and applications. Nanomedicine.

[10] Pankhurst Q.A., Thanh N.T.K., Jones S.K., Dobson J. (2009). Progress in applications of magnetic nanoparticles in biomedicine. J. Phys. D Appl. Phys..

[11] Vallabani N.V.S., Singh S. (2018). Recent advances and future prospects of iron oxide nanoparticles in biomedicine and diagnostics. 3 Biotech.

[12] Dykman L., Khlebtsov N. (2012). Gold nanoparticles in biomedical applications: Recent advances and perspectives. Chem. Soc. Rev..

[13] Kennedy L.C., Bickford L.R., Lewinski N.A., Coughlin A.J., Hu Y., Day E.S., West J.L., Drezek R.A. (2011). A New Era for Cancer Treatment: Gold-Nanoparticle-Mediated Thermal Therapies. Small.

[14] Abadeer N.S., Murphy C.J. (2016). Recent Progress in Cancer Thermal Therapy Using Gold Nanoparticles. J. Phys. Chem. C.

[15] Martín M., Carmona F., Cuesta R., Rondón D., Gálvez N., Domínguez-Vera J.M. (2014). Artificial Magnetic Bacteria: Living Magnets at Room Temperature. Adv. Funct. Mater..

[16] Carmona F., Martín M., Gálvez N., Dominguez-Vera J.M. (2014). Bioinspired magneto-optical bacteria. Inorg. Chem..

[17] Gunn J.S., Bakaletz L.O., Wozniak D.J. (2016). What’s on the Outside Matters: The Role of the Extracellular Polymeric Substance of Gram-negative Biofilms in Evading Host Immunity and as a Target for Therapeutic Intervention. J. Biol. Chem..

[18] Ciszek-Lenda M. (2011). Review paper <br> Biological functions of exopolysaccharides from probiotic bacteria. Cent. Eur. J. Immunol..

[19] González A., Garcés V., Sabio L., Velando F., López-Haro M., Gálvez N., Calvino J.J., Dominguez-Vera J.M. (2019). Optical and tomography studies of water-soluble gold nanoparticles on bacterial exopolysaccharides. J. Appl. Phys..

[20] Martín M., Rodríguez-Nogales A., Garcés V., Gálvez N., Gutiérrez L., Gálvez J., Rondón D., Olivares M., Dominguez-Vera J.M. (2016). Magnetic study on biodistribution and biodegradation of oral magnetic nanostructures in the rat gastrointestinal tract. Nanoscale.

[21] Garcés V., Rodríguez-Nogales A., González A., Gálvez N., Rodríguez-Cabezas M.E., García-Martin M.L., Gutiérrez L., Rondón D., Olivares M., Gálvez J. (2018). Bacteria-Carried Iron Oxide Nanoparticles for Treatment of Anemia. Bioconjug. Chem..

[22] Ramanathan R., O’Mullane A.P., Parikh R.Y., Smooker P.M., Bhargava S.K., Bansal V. (2011). Bacterial Kinetics-Controlled Shape-Directed Biosynthesis of Silver Nanoplates Using Morganella psychrotolerans. Langmuir.

[23] Bansal V., Bharde A., Ramanathan R., Bhargava S.K. (2012). Inorganic materials using “unusual” microorganisms. Adv. Colloid Interface Sci..

[24] Sene S., Marcos-Almaraz M.T., Menguy N., Scola J., Volatron J., Rouland R., Grenèche J.-M., Miraux S., Menet C., Guillou N. (2017). Maghemite-nanoMIL-100(Fe) Bimodal Nanovector as a Platform for Image-Guided Therapy. Chem.

[25] Kolosnjaj-Tabi J., Javed Y., Lartigue L., Volatron J., Elgrabli D., Marangon I., Pugliese G., Caron B., Figuerola A., Luciani N. (2015). The One Year Fate of Iron Oxide Coated Gold Nanoparticles in Mice. ACS Nano.

[26] Kolosnjaj-Tabi J., Marangon I., Nicolas-Boluda A., Silva A.K.A., Gazeau F. (2017). Nanoparticle-based hyperthermia, a local treatment modulating the tumor extracellular matrix. Pharmacol. Res..

[27] Gandouzi I., Tertis M., Cernat A., Saidane-Mosbahi D., Ilea A., Cristea C. (2019). A Nanocomposite Based on Reduced Graphene and Gold Nanoparticles for Highly Sensitive Electrochemical Detection of Pseudomonas aeruginosa through Its Virulence Factors. Materials.

[28] Massart R. (1981). Preparation of aqueous magnetic liquids in alkaline and acidic media. IEEE Trans. Magn..

[29] McFarland A.D., Haynes C.L., Mirkin C.A., Van Duyne R.P., Godwin H.A. (2004). Color My Nanoworld. J. Chem. Educ..

[30] Nikoobakht B., El-Sayed M.A. (2003). Preparation and Growth Mechanism of Gold Nanorods (NRs) Using Seed-Mediated Growth Method. Chem. Mater..

[31] Jin Y., Deng J., Liang J., Shan C., Tong M. (2015). Efficient bacteria capture and inactivation by cetyltrimethylammonium bromide modified magnetic nanoparticles. Colloids Surf. B Biointerfaces.

[32] Pelaz B., Grazu V., Ibarra A., Magen C., del Pino P., de la Fuente J.M. (2012). Tailoring the Synthesis and Heating Ability of Gold Nanoprisms for Bioapplications. Langmuir.

[33] Axelsson L., Ahrné S. (2000). Lactic Acid Bacteria. Applied Microbial Systematics.

[34] Saha K., Agasti S.S., Kim C., Li X., Rotello V.M. (2012). Gold Nanoparticles in Chemical and Biological Sensing. Chem. Rev..

[35] Li N., Zhao P., Astruc D. (2014). Anisotropic Gold Nanoparticles: Synthesis, Properties, Applications, and Toxicity. Angew. Chem. Int. Ed..

[36] Jans H., Huo Q. (2012). Gold nanoparticle-enabled biological and chemical detection and analysis. Chem. Soc. Rev..

[37] Wang S., Lawson R., Ray P.C., Yu H. (2011). Toxic effects of gold nanoparticles on Salmonella typhimurium bacteria. Toxicol. Ind. Health.

[38] Pezzi L., Pane A., Annesi F., Losso M., Guglielmelli A., Umeton C., De Sio L. (2019). Antimicrobial Effects of Chemically Functionalized and/or Photo-Heated Nanoparticles. Materials.

[39] Shareena Dasari T.P., Zhang Y., Yu H. (2015). Antibacterial Activity and Cytotoxicity of Gold (I) and (III) Ions and Gold Nanoparticles. Biochem. Pharmacol. Open Access.

