# Supplementary material for: Magnetic and Golden Yogurts. Food as a Potential Nanomedicine Carrier
Source: Materials (Basel). 2020 Jan 19;13(2):481. doi: 10.3390/ma13020481 (PMC7014100; doi:10.3390/ma13020481)
Supplement: Supplementary file 1 [file materials-13-00481-s001.zip › materials-685727l-SM-proof.docx]

Supplementary Material

Magnetic and Golden Yogurts. Food as a Potential Nanomedicine Carrier

Víctor Garcés, Ana González, Laura Sabio, Carmen M. Sánchez-Arévalo, Natividad Gálvez and José M. Dominguez-Vera *

Departamento de Química Inorgánica, Instituto de Biotecnología, Facultad de Ciencias, Universidad de Granada, 18071 Granada, Spain

***** Correspondence: josema@ugr.es


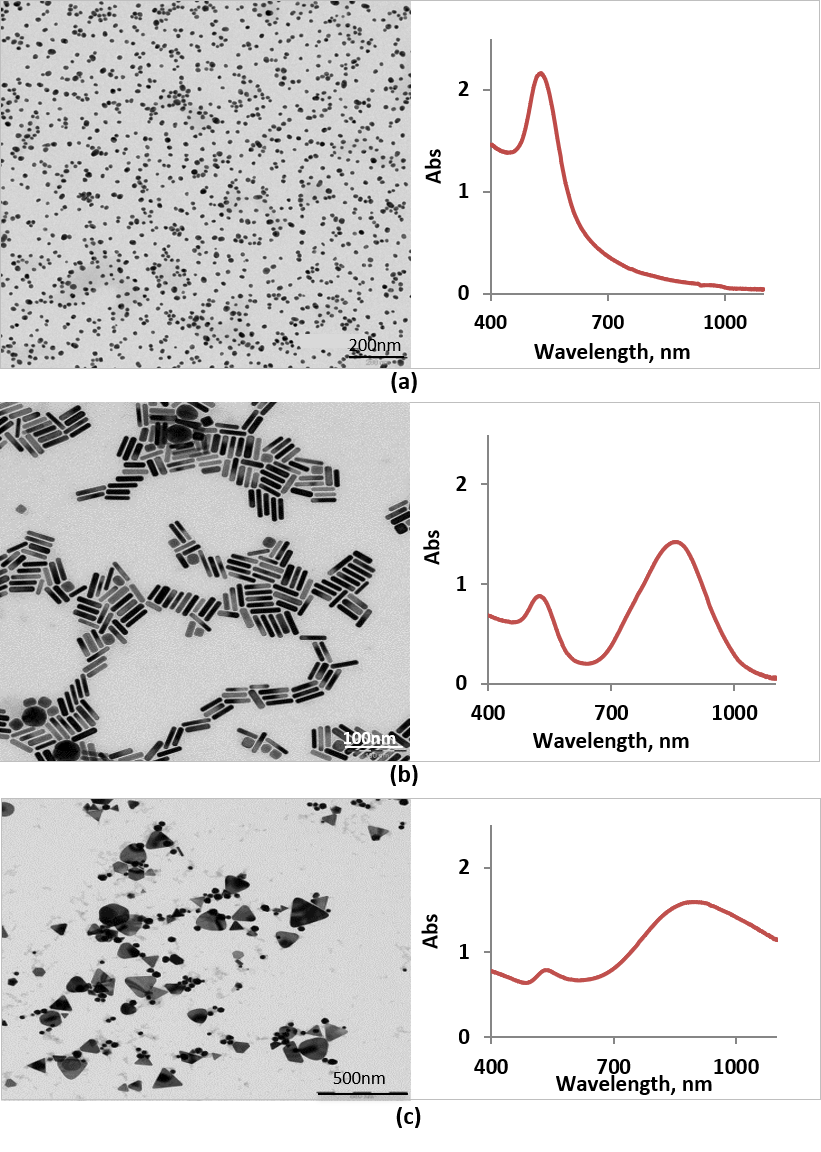


**Figure S1.** TEM images of AuNPs before adhesion to bacteria and their typical SPR bands in the UV-vis spectra. (**a**) AuNSs; (**b**) AuNRs and (**c**) AuNPRs. .

**Video S1.** Movement of the liquid magnetic yoghourt attracted by a 1.2 T magnet. Video File.

| 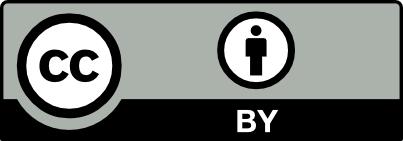 | © 2020 by the authors. Submitted for possible open access publication under the terms and conditions of the Creative Commons Attribution (CC BY) license (http://creativecommons.org/licenses/by/4.0/). |
| --- | --- |
